# Dengue vaccine rollout in India: lessons for Pakistan’s public health preparedness

**DOI:** 10.1097/MS9.0000000000003585

**Published:** 2025-07-16

**Authors:** Santosh Sah, Areeba Ahsan, Rabeea Tariq, Tularam Yadav

**Affiliations:** a Internal Medicine, Dow University of Health Sciences (DUHS), Karachi, Pakistan; b Primary Healthcare Centre (PHCC) Jhorahat, Gramthan, Koshi, Nepal

**Keywords:** dengue, India, Pakistan, TAK-003 (Qdenga), vaccine

## Abstract

Dengue fever continues to challenge public health systems across South Asia, with Pakistan and India facing recurrent outbreaks marked by rising case numbers and hospitalizations. While conventional approaches, such as vector control and public awareness campaigns, have had limited long-term success, vaccines now offer a promising complement to existing strategies. India’s recent approval of the TAK-003 (Qdenga) dengue vaccine marked a turning point in regional prevention efforts. Through strategic partnerships, tiered pricing, improved cold chain infrastructure, and the use of seroprevalence data to guide rollout, India has developed a framework that balances innovation with accessibility. In contrast, Pakistan’s dengue response remains largely reactive, with critical gaps in surveillance, diagnostic capacity, and institutional readiness. Drawing lessons from India’s experience, this article highlights key areas for policy improvement in Pakistan. These include investing in vaccine delivery infrastructure, streamlining regulatory processes, strengthening technical advisory bodies and promoting transparent communication to build public trust. Integrating school-based immunization programs and mobile outreach for underserved populations can ensure equitable access. With a phased, evidence-based approach rooted in local epidemiology, Pakistan has the opportunity to incorporate dengue vaccination into its broader public health strategy, reducing disease burden and increasing preparedness for future outbreaks.

## Introduction

Dengue fever, caused by the single-stranded RNA dengue virus (DENV) of the Flavivirus genus (family Flaviviridae), comprises four distinct serotypes (DEN-1, DEN-2, DEN-3 and DEN-4) and ten genotypes classified on the basis of envelope gene sequence variability^[^1^]^. The clinical presentation of dengue ranges from asymptomatic or mild illness to severe, potentially life-threatening disease. In South Asia, dengue poses an escalating public health threat. The increasing incidence of dengue and the expansion of Aedes mosquito populations are driven primarily by rapid population growth, urban migration, high-density settlements, unplanned urbanization with deficient water and waste infrastructure, climate change, and fragmented, under resourced mosquito control efforts^[^2^]^. Both India and Pakistan have experienced increasingly severe dengue outbreaks, with increasing hospitalizations and mortality^[^3,4^]^. According to the National Institute of Health, Pakistan reported a total of 21 016 confirmed cases of dengue, mostly after the monsoon season, while in 2024, the number rose to 28 427 nationwide.^[^3^]^ India reported 289 235 cases in 2023 and over 233 000 cases in 2024, with peaks typically occurring in October^[^4^]^. Traditional methods, including vector control and public education, have yielded limited long-term effectiveness. In this context, vaccines have emerged as a critical tool to complement existing strategies and reduce the disease burden. Despite decades of research, by early 2025, only two dengue vaccines have been licensed, and neither has seen broad implementation. The recent approval of Takeda’s TAK-003 (Qdenga) vaccine in India represents a pivotal shift in regional dengue prevention efforts^[^5^]^. For Pakistan, which has not yet implemented a dengue vaccination program, India’s experience provides timely and actionable insights. We ensure that our article is compliant with the TITAN Guidelines 2025—governing declaration and use of AI^[^6^]^.

## Materials and methods

### India’s progress

India’s recent steps toward introducing a dengue vaccine offer valuable insights for Pakistan. A key example is the collaboration between Takeda Pharmaceuticals and Biological E., a partnership that not only strengthens local vaccine production but also ensures regional accessibility. This initiative aligns with India’s broader “Make-in-India” campaign and reflects a commitment to bolstering domestic healthcare innovation^[^5^]^. Equally noteworthy is India’s adoption of a tiered pricing model for Qdenga, Takeda’s dengue vaccine. By structuring pricing to accommodate different income groups, the strategy aims to make immunization accessible across socioeconomic strata. Takeda emphasized that one of its primary goals is to ensure that those most at risk of dengue, regardless of income, can benefit from vaccination^[^7^]^. In addition to equitable pricing, India has invested significantly in the cold chain infrastructure required for vaccine storage and transport, as well as systems to monitor adverse events following immunization (AEFI). These measures not only improve the efficiency of vaccine rollout but also help build public confidence in the program. The introduction of Qdenga is set to occur across both public and private healthcare sectors, with a particular focus on pediatric populations under India’s National Immunization Program^[^5^]^. Another strength of India’s approach is its use of seroprevalence data to guide deployment. By identifying regions with high transmission rates, health authorities can strategically target vaccination efforts, maximizing both impact and resource efficiency. If Pakistan was to conduct similar studies, it could develop data-driven, region-specific vaccine strategies aligned with local disease patterns. As emphasized by the WHO, integrating vaccination with effective surveillance and vector control is essential for long-term, sustainable dengue prevention in endemic regions^[^8^]^.

### Gaps in Pakistan’s current dengue response

Despite facing repeated dengue outbreaks, Pakistan has yet to establish a comprehensive national vaccination program against the disease. The country’s response continues to be largely reactive, with vector control measures often delayed, disjointed, and unevenly implemented. In cities such as Karachi, for example, late and inconsistent fumigation campaigns have done little to contain the spread of mosquito-borne illnesses, revealing persistent inefficiencies in the ability of the public health system to manage outbreaks^[^9^]^. Surveillance efforts also fall short. In Rawalpindi, although a considerable number of outdoor vector surveillance sites were inspected, a high percentage remained positive for dengue vectors, underscoring significant gaps in monitoring and control^[^10^]^. On the diagnostic front, the limited laboratory capacity in terms of both the technical infrastructure and funding continues to hinder timely and accurate detection of dengue. A recent study from Karachi highlighted how cost barriers and insufficient diagnostic resources often prevent proper confirmation of suspected cases, complicating disease tracking and patient care^[^11^]^. Moreover, there is a noticeable lack of locally driven research exploring vaccine acceptability, cost effectiveness, and the prevalence of different dengue virus serotypes. The institutional frameworks necessary for assessing and regulating new vaccines remain underdeveloped. The National Immunization Technical Advisory Group (NITAG), which should play a central role in informing vaccine policy, has thus far had minimal involvement in dengue prevention. According to the World Health Organization, Pakistan urgently needs to strengthen its integrated vector management strategies and build more robust, responsive surveillance systems tailored to its specific epidemiological landscape^[^12^]^.

## Results and discussion

### What Pakistan can learn from India

India’s example offers several valuable lessons for Pakistan. India’s dengue vaccine rollout provides critical insights for Pakistan, emphasizing the need for operational readiness, regulatory strengthening, and early public engagement. Utilizing existing infrastructure and ensuring transparent communication can enable efficient vaccine deployment. The following key strategic priorities can be adopted for dengue vaccine preparedness in Pakistan [Table 1].

**Table 1:** Key Strategic Priorities for Dengue Vaccine Preparation in Pakistan

Priority Area	Recommendations
Operational Readiness	Invest in cold chain infrastructure
	Strengthen AEFI monitoring
	Train immunization workforce
Regulatory Capacity	Streamline vaccine evaluation processes
	Fast-track WHO-prequalified vaccines
	Strengthen DRAP and NITAG
Public Trust & Communication	Launch transparent communication campaigns
	Address vaccine hesitancy
	Engage communities and civil society
Regional Collaboration	Coordinate cross-border surveillance and data sharing
	Participate in collaborative clinical trials
	Utilize WHO-SEARO and SAARC platforms

AEFI: adverse event following immunization, DRAP: Drug Regulatory Authority of Pakistan, NITAG: National Immunization Technical Advisory Group, SAARC: South Asian Association for Regional Cooperation, WHO: World Health Organization, WHO-SEARO: World Health Organization–Southeast Asia Regional Office.

This table outlines strategic priorities for dengue vaccine preparedness in Pakistan, drawing on lessons from India’s implementation of the TAK-003 vaccine. It highlights key actions across operational, regulatory, public engagement, and regional cooperation domains that are essential for effective vaccine deployment.

### Policy recommendations

The development of a dengue vaccination has taken almost five decades, with difficulties including inadequate knowledge of the immune responses required for protection and a lack of a suitable animal model. Starting a systematic policy debate in Pakistan is crucial for evaluating the viability and timing of dengue vaccination acceptance as vaccine technology develops. Learning from India’s recent Qdenga® vaccination rollout, Pakistan can implement a phased, data-driven plan aimed at high-burden regions^[^13^]^. By means of mapping dengue hotspots and identifying at-risk populations, one can maximize resource allocation and vaccination distribution. Making wise decisions depends on Pakistan strengthening its National Immunization Technical Advisory Group (NITAG). Vaccine safety, suitability, and effectiveness should be independently evidenced-based reviews performed by NITAG, specifically for the local setting. This guarantees a trustworthy, open policy direction consistent with the National Immunization Program (NIP). Public trust and vaccine acceptance are vital for success. School-based immunization programs targeting adolescents, complemented by community engagement, risk communication, and behavioral research, can drive uptake^[^14^]^. For out-of-school youth, mobile health teams and community clinics should be mobilized. Lessons from Pakistan’s COVID-19 vaccine rollout demonstrate the importance of accessibility and coordinated outreach. To bolster national preparedness, regional collaboration is also key^[^15^]^. Platforms such as the South Asian Association for Regional Cooperation (SAARC) and the World Health Organization–South East Asia Regional Office (WHO-SEARO) offer opportunities for joint surveillance, data sharing, vaccine procurement, and outbreak response. Learning from neighboring countries such as India can strengthen Pakistan’s strategies and foster harmonized responses to transboundary health threats^[^16^]^. By empowering the NITAG, leveraging existing immunization infrastructure, and promoting regional partnerships, Pakistan can take timely action to integrate dengue vaccines into public health planning and prevent future outbreaks.

## Conclusion

To prevent future dengue outbreaks in Pakistan, immediate policy action is needed. Dengue immunization must be included in national public health plans as soon as possible because of the increasing frequency and severity of the disease. Pakistan can learn from India’s planned deployment by implementing a phased strategy backed by stronger institutions such as NITAG and supported by evidence. Coordinated surveillance, data sharing, and response planning necessitate regional cooperation through organizations such as WHO-SEARO and SAARC. Pakistan can better prepare for dengue and other transboundary public health challenges by drawing on these collaborations and experiences.

## Data Availability

No datasets were created.
